# Protein Unfolding in Freeze Frames: Intermediate States are Revealed by Variable-Temperature Ion Mobility–Mass Spectrometry

**DOI:** 10.1021/acs.analchem.2c03066

**Published:** 2022-08-24

**Authors:** Jakub Ujma, Jacquelyn Jhingree, Emma Norgate, Rosie Upton, Xudong Wang, Florian Benoit, Bruno Bellina, Perdita Barran

**Affiliations:** Michael Barber Centre for Collaborative Mass Spectrometry, Manchester Institute of Biotechnology, University of Manchester, 131 Princess Street, Manchester M1 7DN, United Kingdom

## Abstract

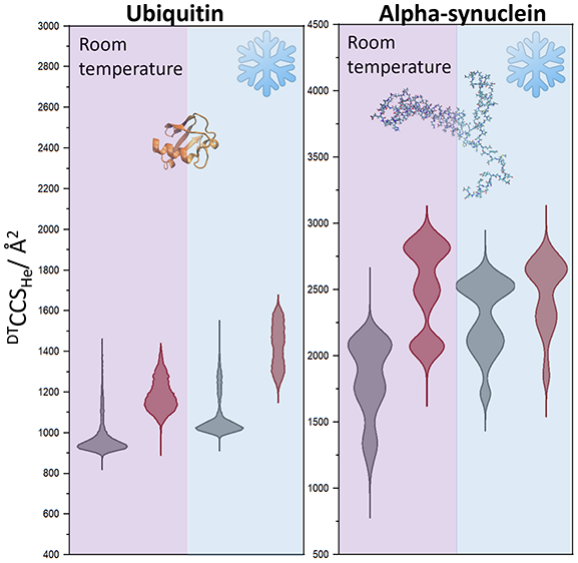

The gas phase is an idealized laboratory for the study of protein structure, from which it is possible to examine stable and transient forms of mass-selected ions in the absence of bulk solvent. With ion mobility–mass spectrometry (IM-MS) apparatus built to operate at both cryogenic and elevated temperatures, we have examined conformational transitions that occur to the monomeric proteins: ubiquitin, lysozyme, and α-synuclein as a function of temperature and in source activation. We rationalize the experimental observations with a temperature-dependent framework model and comparison to known conformers. Data from ubiquitin show unfolding transitions that proceed through diverse and highly elongated intermediate states, which converge to more compact structures. These findings contrast with data obtained from lysozyme—a protein where (un)-folding plasticity is restricted by four disulfide linkages, although this is alleviated in its reduced form. For structured proteins, collision activation of the protein ions in-source enables subsequent “freezing” or thermal annealing of unfolding intermediates, whereas disordered proteins restructure substantially at 250 K even without activation, indicating that cold denaturation can occur without solvent. These data are presented in the context of a toy model framework that describes the relative occupancy of the available conformational space.

The measurement of folding and unfolding landscapes of proteins is a scientific question that has intrigued experimentalists for more than 100 years, and yet even for the most studied model proteins such as ubiquitin, there remain unanswered questions regarding the conformations adopted on route to the folded state.^1−6^ In solution, ubiquitin folding exhibits two-state kinetics, but some studies suggest the presence of additional on- or off-pathway intermediate states.^7,8^ A compact native N-state and a partially folded A-state have been identified in different solution conditions, as well as in gas-phase studies,^1,9^ but reversible, conformational transitions have been induced by altering solution conditions,^10−12^ pressure,^13^ and temperature.^12,14^ Spectroscopic methods including time-resolved NMR^7^ and IR experiments can be used collectively to reveal intermediate states occurring on a microsecond to second timescale,^8^ but the intrinsic characteristic of these in solution methods means the output is spatially averaged. While time-resolved information is indispensable for kinetic analyses, it does not readily enable concomitant information on the intermediates’ structure. Such structural resolution of intermediates in a restructuring pathway is even more problematic with less ordered proteins, which will present a wide array of interconverting conformers and a far shallower potential energy surface. To demonstrate the potential of variable-temperature ion mobility–mass spectrometry (VT-IM-MS) in studying fast structural transition, with unparalleled resolution, we apply it to three well-studied proteins and show how it can be used to determine folding transitions and transient intermediates.

IM-MS allows the measurements of collision cross section (CCS) distributions which inform on the conformational preferences of a given molecule and can be readily compared with bulk phase measurements or with those from calculated candidate geometries with utility for both structured and unstructured proteins.^17,15,16^ Using IM-MS, several groups have investigated conformational preferences of gaseous ubiquitin ions at ambient temperatures, with good evidence for the preservation of the N-state.^9,18−21^ It has been reported that increased buffer gas temperature and/or collisional activation can lead to elongation events,^22^ attributed to unfolding pathways.^23,24^ Similarly, gas-phase compaction following charge reduction has been demonstrated by Valentine et al.^25^ and more recently by Laszlo et al.^26^ These elongation/compaction phenomena appear substantially step-wise, conceptually resembling mechanically induced unfolding experiments performed by atomic force microscopy.

Solution- and gas-phase behavior of lysozyme contrasts that of ubiquitin. It is approximately twice the size of ubiquitin with 129 amino acids, and due to its four intramolecular disulfide bonds,^27^ it often exists in highly stable folds both in solution and gas phases.^27−29^ In solution, the folding pathway of lysozyme is typically reported as a two-state kinetic transition^30^ between the native N-state and the denatured helical H-state and kinetically trapped, partially folded transient intermediates may be possible.^31,32^

Here, we show how variable-temperature ion mobility–mass spectrometry experiments on a high-resolution instrument^33^ can uniquely delineate unfolding intermediates and conformational landscapes of proteins. We contrast the behavior of the structured protein ubiquitin with lysozyme in both disulfide intact and reduced forms and with the intrinsically disordered protein α-synuclein. The key benefits of this approach are the ability to freeze the metastable transition states, assess their size, and compare these to that of the “native” and “unfolded” forms as well as to all intermediates.

## Experimental Section

### Samples

Ubiquitin (from bovine erythrocytes) and lysozyme (from chicken egg white) were purchased from Sigma-Aldrich (Poole, U.K.). Human recombinant α-synuclein was a gift from Rajiv Bhat, expressed in BL21 (DE3) *Escherichia coli* and purified as described previously.^34^ Ammonium acetate was purchased from Fisher Scientific (Loughborough, U.K.). Final protein concentrations were prepared to 50 μM ubiquitin, 30 μM lysozyme, and 20 μM α-synuclein all in 50 mM ammonium acetate, pH 6.8. Disulfide-reduced lysozyme was prepared by adding 10 mM DTT (Fluorochem Ltd., U.K.) to the 30 μM lysozyme solution and left to incubate at 30 °C overnight. Full disulfide reduction was verified by observing a mass shift of +∼8 Da (*m*/*z* 1790.2 for 8+ charge state) compared to intact (*m*/*z* 1789.1 for 8+ charge state).

### VT-IM-MS

Our main experimental arrangement and measurement principles have been described in detail elsewhere.^33^ Briefly, the ions are created in a nano-ESI source (capillary set to 1.0–1.4 kV), then transferred *via* two ion guides into the variable-temperature (VT) IM cell (50.5 cm). The pressure in the ions guides is 0.5 and 1.5 Torr and in the drift cell 2.0 Torr helium; pressure in each region is recorded throughout the experiment using baratrons. The temperature of the background gas in the first ion guide is close to 300 K, maintained that way since the chamber is in contact with air, whereas the temperature of the drift gas (inside the VT-IM cell) can be varied between 150 and 500 K using a heating and cooling jacket. Ion activation is induced by increasing the voltage offset (0–70 V) between the two ion guides. The ions are activated by collisions with residual air from the source at ∼300 K; importantly, this is largely independent of the temperature of the gas inside the VT-IM cell (160–300 K). Following activation, ions will experience thermalizing collisions as they move through the second ion guide and into the VT compartment. Ions are then accumulated inside the ion buncher and pulsed into the VT drift region where the mobility separation takes place.

Inside the VT chamber, the ions acquire the temperature of the helium gas while being accumulated; upon release, the ions travel through the VT drift region where the mobility separation takes place. Compact structures drift faster than the extended forms of the same charge state. In such IM-MS experiments, *m*/*z* data obtained for each ion has an associated arrival time distribution (ATD), which can be then converted to a collision cross section distribution.^33,35^ To rule out the possibility of charge stripping during activation, mass-selected IM-MS of ubiquitin 6+ and 7+ and intact lysozyme 8+ and 9+ were recorded at a range of increasing activation energies (see Figures S1A,B and S2A,B, respectively).

### Ubiquitin

**Figure 1:**
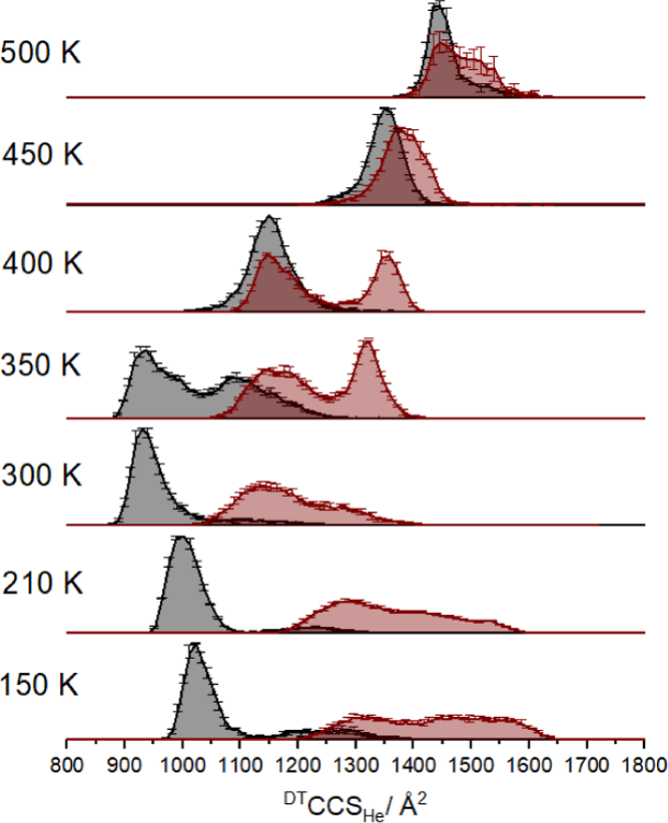
Collision cross section distributions of ubiquitin 6+ sprayed from 50 μM solution in 50 mM ammonium acetate, pH 6.8. Data obtained with no in-source activation (dark gray distributions) and in-source activation of 70 V (red distributions) are shown at a range of temperatures. Error bars correspond to standard deviation from three, 1 min long acquisitions. Data have been normalized to the area under the dark gray (i.e., nonactivated) traces.

Figure 1 shows the collision cross section distributions obtained for 6+ ubiquitin ions between 150 and 500 K with and without in-source activation (red and dark gray traces, respectively). The corresponding ^DT^CCS_He_ distributions and mass spectra for the 5+ and 7+ charge states are presented in Figures S2–S4. At ambient temperature (300 K), nonactivated ions present as a compact population around 950 Å^2^, consistent with the reported literature data.^21,36^ Upon reducing the temperature, the ^DT^CCS_He_ of the compact population shifts to ∼1050 Å^2^ at 150 K. We attribute this increase to the expected dependence of long-range ion–molecule interactions rather than a conformational transition (see the Supporting Information for further details).^37^ At 350 K and above, several conformational transitions are observed for nonactivated ions.

When the VT compartment is held at sub-ambient temperatures (lower 2 traces of Figure 1), the internal temperature of the collisionally activated ions is rapidly reduced; this may lead to kinetic trapping of energetic conformational states. Data obtained at sub-ambient temperatures indicates that these forms can be, to a large extent, preserved on the measurement timescale. When the IM cell is held at ambient or elevated temperatures, collisionally activated ions may refold during accumulation and, to a lesser extent during separation in the drift region. The typical drift times are between 4 and 12 ms and can be altered by changing the applied electric field. No apparent change is seen in these ^DT^CCS_He_ distributions as a function of drift voltage, which implies that structural rearrangements occur primarily during accumulation.

Tuning the collisional activation conditions allows refolding intermediates to be captured in the low-temperature drift tube, and this evolution as a function of activation voltage is shown in the CCS distributions (Figure S6). At room temperature, the ^DT^CCS_He_ profiles of the activated ions lie between 1100 and 1400 Å^2^, whereas at 150 K, activation produces significantly more extended forms with (^DT^CCS_He_ 1250–1650 Å^2^). The most extended state achieved at 150 K (1650 Å^2^) aligns with the predicted 150 K ^DT^CCS_He_ of the most extended state we observe at 500 K (using the PSA method). This highly extended form has not refolded on the timescale of the experiment in the low temperature of the drift tube; numerous lower CCS intermediate states are observed. Ion–molecule interactions may be different for elongated ions than for the compact forms; nevertheless, the magnitude of the change and difference in the shape of the distribution cannot be explained solely by ion–molecule interaction potentials.^38^ At elevated temperatures (above 150 K), extended intermediates “frozen out” at 150 K (1300–1600 Å^2^) can refold to adopt temperature-specific conformations (e.g., 1150 Å^2^ at 300 K). At 350 K and above, the conformational profiles of the activated ubiquitin ions are significantly different from the low-T profiles. At 400 K, we observe two distinct populations of apparently equal stability, both of which may derive from different annealing pathways from the extended activated precursor. At 450 and 500 K, more extended forms dominate the ^DT^CCS_He_ distributions which are also narrower, suggestive of a reduction in the number of conformations present.

Considering the data obtained for ubiquitin, a structurally flexible protein, we conclude that the transition from the initial “compact” ions (denoted N-state, ∼1090 Å^2^) is irreversible on our experimental timescales – thus either the energy of this state is higher in the gas phase or that the barrier between this and the intermediates and extended states is elevated in the absence of solution. Following activation, unfolding proceeds through a variety of intermediates and transition states which at room temperature converge to structures at 1100–1400 Å^2^. At cryogenic temperatures, we can slow down the interconversion process and perhaps kinetically trap those metastable species, which results in a broad CCS profile (Figure 1, 210–150 K). We hypothesize that ubiquitin N 6+ undergoes an inside-out transition, which may involve both self-solvating of “native protonation sites” and accommodating for the coulombic repulsions.

### Lysozyme

**Figure 2:**
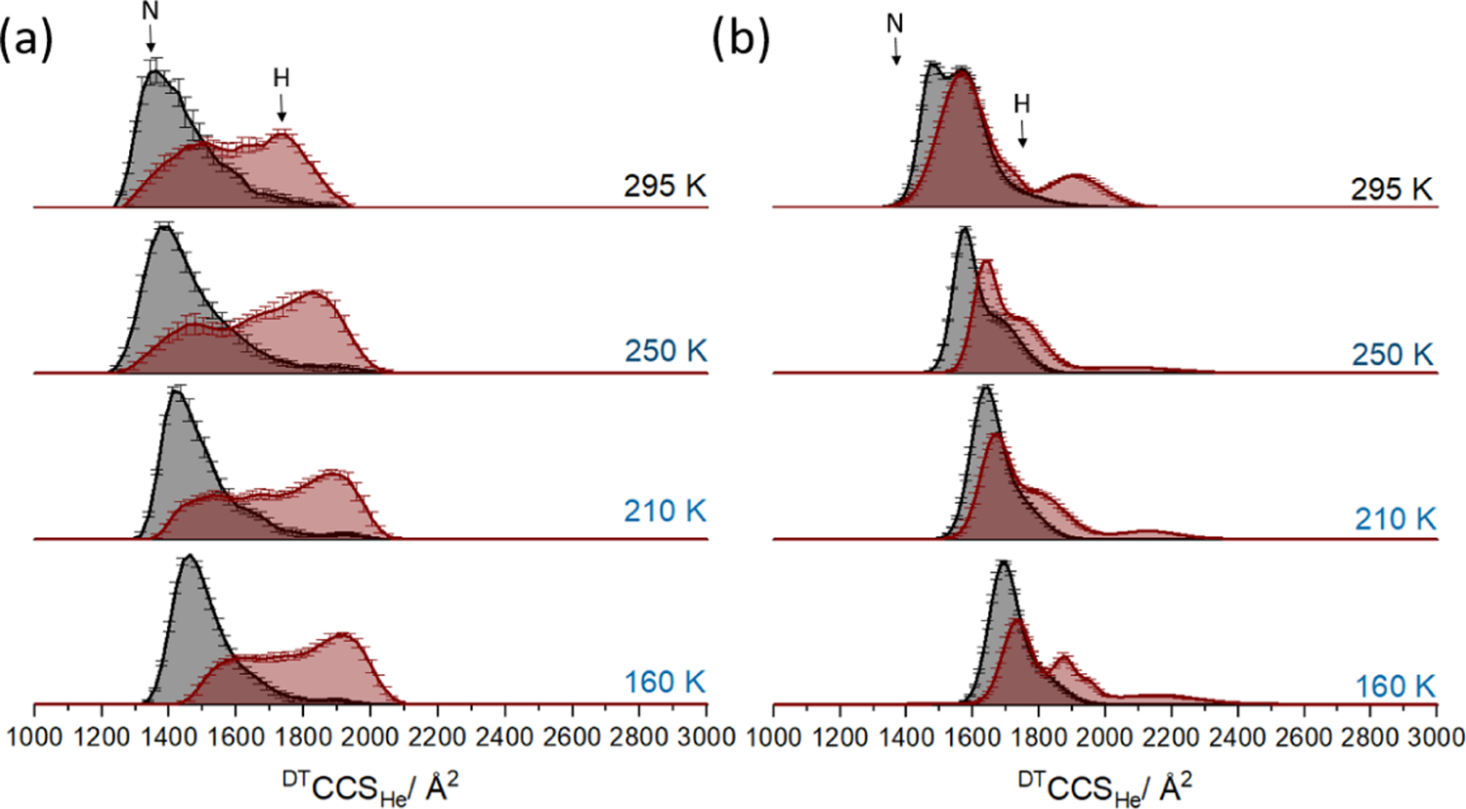
Collision cross section distributions of intact (a) and disulfide-reduced (b) lysozyme 8+ (30 μM in aqueous 50 mM ammonium acetate, pH 6.8). Disulfide-reduced lysozyme additionally contains 10 mM DTT and was incubated at 30 °C overnight. Dark gray CCS distributions represent data obtained with no in-source activation at a range of temperatures (160–295 K). Red CCS distributions represent the respective data recorded with in-source activation (voltage offset of 90 V). In part (a), N- and H-states are indicated. In part (b), black arrows refer to the more compact conformer and gray arrows refer to the more extended conformer for the activated measurements. Error bars correspond to standard deviation from six, 30 s long acquisitions. Activated data has been normalized to the area of the nonactivated data.

We performed similar measurements on the disulfide intact and reduced forms of the protein lysozyme focusing here only on temperatures at or below ambient; we have previously reported high-temperature data for this protein, and as for ubiquitin, lysozyme thermally denatures as the temperature of the drift gas is increased (Figure S8). At 300 K, lysozyme 8+ charge state presents a ^DT^CCS_He_ of ∼1300 Å^2^ and there is a small presence of a more unfolded conformer at ∼ 1800 Å^2^. At 360 K, the conformer at ∼1800 Å^2^ dominates suggesting a thermally induced shift to the more unfolded state (Figure S8).^32^ Lysozyme charge states 10+ and 11+ demonstrate a shift of ^DT^CCS_He_ from ∼1900 Å^2^ at 300 K to ∼2500 Å^2^ at 360 K. Here, Figure 2 shows the ^DT^CCS_He_ distributions for the 8+ ions of lysozyme, both intact (a) and disulfide-reduced (b), between 160 and 295 K; accompanying mass spectra and CCS distributions, nonactivated and activated for 7+ ions can be found in Figures S7–S9. For the 8+ ions, the nonactivated intact species (solid dark gray line) predominantly present as the compact N-state (∼1360 Å^2^ at 295 K).^32^ As for ubiquitin, the ^DT^CCS_He_ increases with decreasing buffer gas temperature (250 K, 1370 Å^2^; 210 K, 1400 Å^2^; 160 K, 1460 Å^2^), which again we attribute to the expected temperature dependence of the ion–molecule interaction.^39^

Following in-source collisional activation (red trace Figure 2), as for ubiquitin tuned for maximum extension (Figure S10), we see an increase in the population of the extended state (∼1750 Å^2^ at 295 K) and the retention of some N-state (native state) population. As we decrease the temperature of the drift cell further, we observe that the population of the H-state (denatured helical state) shifts away from the nonactivated N-state, the effect being most pronounced at 160 K. This indicates that as for ubiquitin, we are able to “freeze” some of the transient intermediates within the timescale of the experiment. Similar behavior is found for the 7+ charge state (Figure S9a), although the available activation energy is not sufficient to substantially populate the H-state.

The nonactivated disulfide-reduced presents a ^DT^CCS_He_ distribution centered on ∼1550 Å^2^, which is around 190 Å^2^ larger than the equivalent ^DT^CCS_He_ distribution for intact lysozyme. This larger structure is attributed to the loss of disulfide bonding leading to less restricted conformations. Again, the ^DT^CCS_He_ increases with decreasing temperature (250 K, 1580 Å^2^; 210 K, 1620 Å^2^; 160 K, 1680 Å^2^), an overall increase of ∼9% over the temperature range. Following collisional activation, a proportion of conformers occupy the more extended state (1920 Å^2^ at 295 K), presenting as a conformer of higher CCS than intact lysozyme (1720 Å^2^ at 295 K). At 250 K, the extended state shifts further away from the nonactivated state, as for ubiquitin, suggesting “freezing” of more unfolded intermediates.

At 210 and 160 K, the occupancy of a more extended conformer for the activated ions increases (2140 Å^2^ at 210 K; 2190 Å^2^ at 160 K), and additionally there is a second conformer (indicated in Figure 2b H-state) at lower ^DT^CCS_He_ which increases in intensity at lower drift gas temperatures.

For both charge states of intact lysozyme (Figures 2a and S9a) the width of the un-activated ^DT^CCS_He_ distribution at 250 K is greater than at 210 K. The CCS distributions at 210 and 160 K indicate more resolved conformers at ∼1650 Å^2^ as well as some N-state. The enhanced resolution is expected – it should scale with √*T*;^40^ the fact that we do not see this for ubiquitin provides additional evidence that following transfer into the gas phase, N-state conformers are readily disrupted. The increased width at 250 K for un-activated lysozyme 8+ has been reported by us previously^32^ (Figure S10) and indicates a temperature-specific transition at around −20 °C, which does not happen at lower, deep-freezing temperatures.

### Extending the Analysis to Other Proteins

We have explored the variation in ^DT^CCS_He_ at lower temperatures with other proteins,^23,32^ including α-synuclein and monoclonal antibodies.^41^Figures S12 and S13 show IM measurements and mass spectra from α-synuclein for 9+, 11+, and 12+ charge states. At room temperature, multiple distinct conformers are present, showing populations centered at ^DT^CCS_He_ ∼1600, ∼1850, and ∼2100 Å^2^, indicative of the intrinsically disordered nature of this protein as previously reported.^42,43^ On activation, as for ubiquitin and lysozyme, these conformers rearrange, but here into more extended states, displaying a distinct increase in ^DT^CCS_He_ of +∼500 Å^2^ for the lower charge states and +∼800 Å^2^ for the highest charge state for activated peaks at 295 K. Conversely at 250 K there is little distinction between distributions for the nonactivated and activated protein; the 11+ charge state centers around ∼2500 Å^2^ and the 12+ centers around ∼2700 Å^2^ for both nonactivated and activated distributions. For nonactivated α-synuclein, the increase in ^DT^CCS_He_ from room temperature to 250 K is larger than we would expect. This suggests at 250 K we are observing structural rearrangement to the larger conformer induced by the temperature at which we take the measurement.

### Deriving a Structural Framework from Experimental Observations

**Figure 3:**
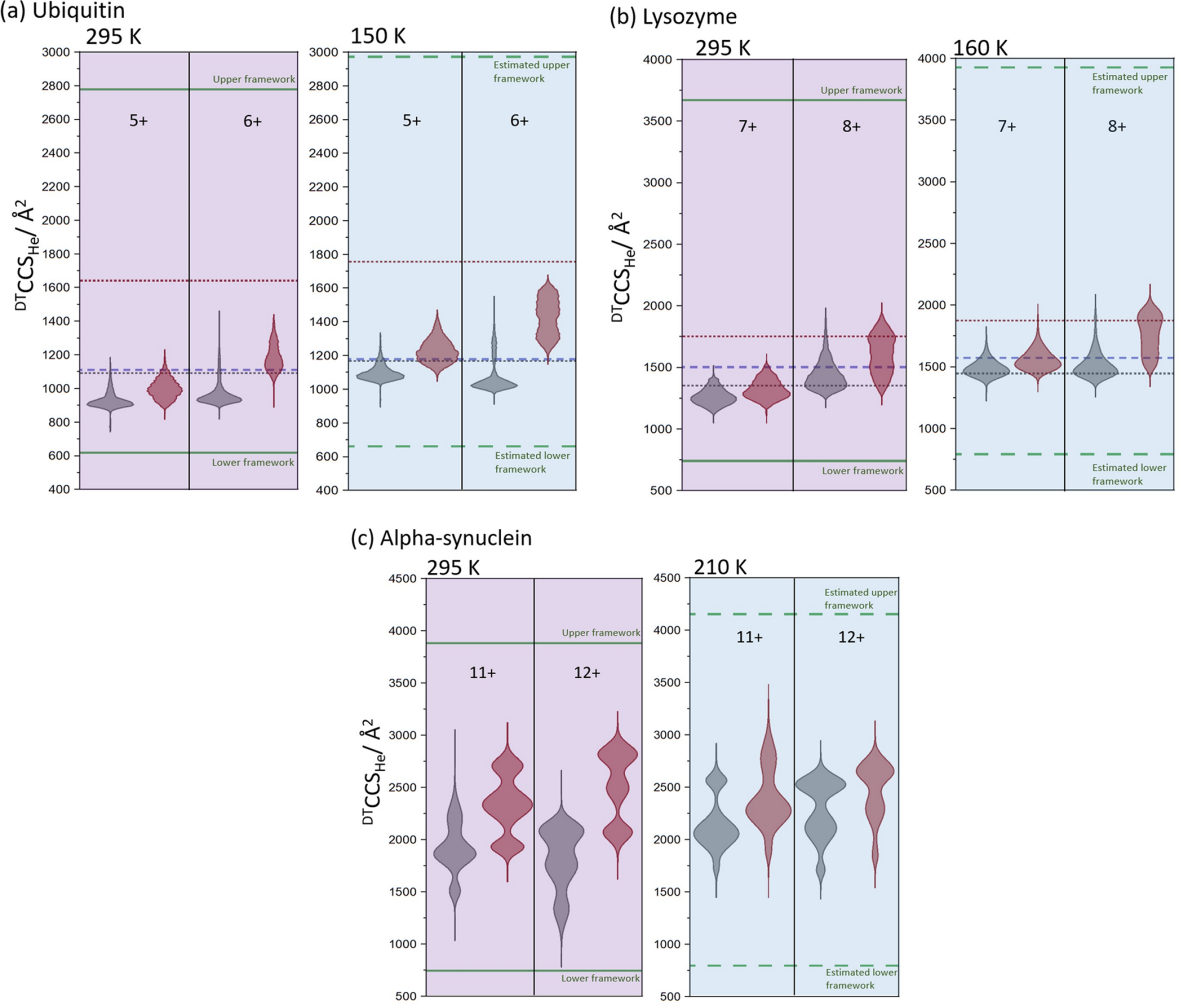
Summary of experimental CCS distributions for ubiquitin 5 and 6+ (a); lysozyme 7 and 8+ (b); and α-synuclein 11 and 12+ (c) for both nonactivated (gray violin plots) and activated (red violin plots). CCS values from pdb structures were calculated at each temperature using Projection Super Approximation method^38,46,51,52^ and are shown as blue dashed lines for ubiquitin (a) and lysozyme (b). Previously published CCS values are shown for N-state (gray dotted line in (a)) and A-state (red dotted line in (a)) of ubiquitin^53^ and for the N-state (gray dotted line in (b)) and H-state (red dotted line in (b)) of lysozyme.^32^ The maximum and minimum CCS is depicted with solid green lines for each protein, calculated using the Framework model^17^ and are scaled from room temperature to the lower temperature in each case (see the Supporting Information for details of framework calculations).

To understand the context of these experimental observations, it is first important to estimate the expected increase in CCS due to the increased effect of the ion buffer gas interaction potential at lower temperatures. To do this, we calculated theoretical ^DT^CCS_He_ from pdb structures of lysozyme (1E8L) and ubiquitin (2RU6) at 295 and 160 K (for lysozyme) and 150 K (for ubiquitin) using the projected superposition approximation (PSA).^44^ For both the change in theoretical ^DT^CCS_He_ across the presented temperature range was found to be ∼6%, in fairly good agreement with that measured experimentally (∼7%). For each of the three proteins investigated, we have plotted the CCS distributions of the nonactivated and activated forms as violin plots to allow a facile comparison of the change in CCS at a function of temperature (Figures 3 and S14). For the structured proteins, we have added lines to indicate the calculated CCS_He_ values found for the N states and for the A (ubiquitin) and H (lysozyme) states. For α-synuclein, no such structure exists. We have also depicted on these plots the boundaries that we predict for these proteins for the most compact and most extended forms, using our framework model.^17,45^ In brief, the most compact form is calculated using the equation for a protein sphere (Supporting Information, eq 1), assuming the protein is in its most folded, globular state. For the most extended form, it is assumed that the protein amino acid sequence is fully extended so the α carbons in the protein chain are as far apart as dictated by the covalent bonding in the polypeptide chain. The CCS of this polypeptide chain is calculated by approximating it as a cylinder (Supporting Information, eq 2), where the side chains are included as beads with the appropriate diameters. We have here scaled these values for the lower-temperature measurements based on PSA predictions^46,47^ and empirical measurements from a body of work using VT-IM-MS to examine the effect of temperature on CCS values^41^ (see the Supporting Information for method details).

These plots allow us to consider the conformational landscapes occupied by these proteins as a function of activation and how these are captured at lower temperatures. For ubiquitin (+6), we conclude that at low temperatures following activation, we kinetically trap metastable intermediate/transition states somewhat smaller than the A state, which are not observable in ambient temperature measurements. These are evident in the altered CCS distributions for both charge states. With lysozyme, it is evident from the violin plot that the H state is accessible following activation, and also that the occupancy of the N-state is retained, in part. At 160 K, there is a significant overlap of activated and nonactivated arrival time distributions. This suggests that the structural motifs in activated ion populations are to a large extent similar to those in nonactivated ions. This correlates with the known, high stability of lysozyme both in solution and gas phases. Disulfide-reduced lysozyme showed much less overlap, with a higher proportion of conformers occupying the unfolded state when activated. For lysozyme and ubiquitin, the experimental observations allow us to consider the free energy surfaces that are explored by proteins as they refold in the gas phase compared with solution (Figure S15).

The intrinsically disordered protein α-synuclein occupies several distinct conformations which cover more than 50% of the possible conformational space (Figures 3c and S12). These distributions alter as the protein is examined at low temperature, where there is both the expected increase as well as a change in the relative population of these conformers (Figures 3c and S12) indicative of a free energy surface that has many closely located minima. On activation, the protein is readily promoted to larger conformers, which again illustrates the vast conformational landscape. We hypothesize that α-synuclein experiences structural rearrangement which we consider to be cold denaturation, at 250 K (Figure S12) as for lysozyme, but to a greater extent due to its disordered nature. Therefore, even the nonactivated ions restructure at 250 K. Intriguingly, there is little difference in the ion mobility behavior between ions which unfold due to activation energy applied in-source (gray dotted lines, Figure S12 250 K) or ions that have undergone structural rearrangement due to the lower temperature (solid black lines, Figure S12 250 K).

We can contemplate whether conformational transitions observed here bear any resemblance to the reversible conformational transitions, exhibited in solution.^14,48^ For Ubiquitin, changes in ^DT^CCS_He_ are to a large extent “reversible” for conformations between 1100 and 1650 Å^2^ while this is not the case for the structures with ^DT^CCS_He_ below 1000 Å^2^. It has been previously observed that ubiquitin adopts intermediate “I-states” (with ^DT^CCS_He_ 1400–1700 Å^2^) which were correlated with a solution-phase-specific A-state.^21^ Extended structures with a ^DT^CCS_He_ of up to 2000 Å^2^ have also been reported and classified as gas-phase-specific conformations or the unfolded U-state.^9,19,21^ We do not observe any “compact intermediates”, which suggests that once activated, they cannot refold to the initial state in the timescale available, which suggest that the U-state is more stable under these conditions. As an additional check, we performed in-source activation at a range of collision energies (Figures S4 and S9); yet again no “compact intermediates” are observed. Therefore, while the initial compact conformers of ubiquitin (∼950 Å^2^) are likely to contain solution-phase-specific secondary structures which can be preserved in the gas phase with very gentle ESI conditions,^21^ the data here shows that all transitions from such solution-specific folds appear “irreversible” on the timescale of our experiment. This is in accord with the original concept described by Smith and Light-Wahl: “Once the structure of the molecular ion has been lost, however, long-range coulombic forces should effectively preclude the reverse process.”^49^ With lysozyme the disulfide bridges appear to retain elements of the solution fold (Figure 3b) even when activated and we can observe restructuring back to the N-state. Lysozyme is more susceptible to cold denaturation at 250 K than ubiquitin which can retain its native fold through lower temperature. α-synuclein is highly plastic with a dramatically different free energy landscape which is readily manipulated in the gas phase.^50^ Performing IM at low temperatures (especially 250 K) causes substantial disruption of this protein irrespective of whether it has been activated, and intriguingly this results in a conformation range that is closer to that found in SAXS measurements that we observe in room temperature IM measurements.^42^ Given the opportunity to manipulate the time and temperature over which we can measure the conformers of proteins this method provides a tantalizing opportunity to map conformational landscapes.

## Summary and Outlook

Data presented here show the unique strengths of VT-IM-MS measurements to determine the conformational landscapes of monomeric proteins. Collectively, our results show that gas-phase restructuring on short experimental timescales (ms) may proceed through highly extended, intermediate states which then converge to “gas-phase compacted” structures. With VT-IM-MS methodology, we can directly visualize the remarkable structural diversity of those states, as previously reported using fragmentation methods, but now with the temperature of the ion defined.^24^ By affecting the temperature at which the refolding occurs, we obtain snapshots of the restructuring processes that occur and remarkably we observe that lysozyme and α-synuclein readily restructure to larger conformers at 250 K without any activation. Such observations would be very hard to achieve with any other method.

Considering that a “compact” state of a protein in the gas phase is itself a kinetically trapped form, for small and flexible proteins these measurements show that we still need to better understand the bias given to solvated ensembles by the electrospray and desolvation process and by the dominance of electrostatics in gas-phase structures. In solution, a thermally induced, reversible conformational transition for ubiquitin has been reported to occur between 330 and 370 K. Moreover, an irreversible transition was found to occur above 400 K.^14^ Although it is difficult to compare the mechanistic details of the thermally induced unfolding in solution with that which occurs in the absence of solvent, our high-temperature data is consistent with these solution-phase findings; however, it remains to be seen whether this is a general effect.

For ubiquitin, our results indicate that a variety of intermediates (some of which are highly elongated) are present in accord with previous work of Breuker et al.^24^ These intermediate forms then appear to rapidly converge to compacted conformational families. We note that only the latter species are observed in ambient temperature IM experiments. The gas-phase folding pathway of nonreduced lysozyme is expectedly more restrained, resembling the two-state solution model. However, cryogenic IM-MS still captures some, partially folded intermediates between the N- and H-states, and when the protein is reduced, more conformational plasticity is obtained. Our data on α-synuclein again reveal the power of IM-MS in the study of disordered proteins, and with all three proteins, is it apparent that with VT-IM-MS, we can sample conformational states which may be inaccessible at room temperature.

Finally, we note that the experiment presented here is analogous to “simulated annealing”, an approach employed frequently in computational simulations of protein structure and where ubiquitin is often used as a benchmark.^3^ In the future, we intend to extend the VT-IM-MS method to allow for the measurement of timescales of the folding events presented here. Given the ease by which the CCS parameter can be predicted for any candidate geometry, we envisage that such time-resolved data could constitute a unique tool for assessing protein structural dynamics.

## Supporting Information Available

Details of experimental setup and data processing, DTCCSHe profiles of 5+ and 7+ ubiquitin and 7+ intact lysozyme charge states, 7+ for disulfide-reduced lysozyme, for 9+, 11+, and 12+ charge states of α-synuclein and accompanying n-ESI mass spectra, and data from ambient IM-MS control experiments (PDF)

## Notes

J.U. and P.B. devised the study. J.U. performed experiments on ubiquitin and analyzed the data. J.J. ran initial experiments on lysozyme (data not shown), and R.U., B.B., and F.B. performed subsequent experiments and all analyses on intact lysozyme. E.N. and X.W. performed disulfide-reduced lysozyme and α-synuclein experiments and analysis including in support of the framework model. J.U. drafted the manuscript with P.B., R.U., and E.N. All authors contributed to its final form.
