# Supplementary material for: Protein Unfolding in Freeze Frames: Intermediate States are Revealed by Variable-Temperature Ion Mobility–Mass Spectrometry
Source: Anal Chem. 2022 Aug 24;94(35):12248–55. doi: 10.1021/acs.analchem.2c03066 (PMC9453741; doi:10.1021/acs.analchem.2c03066)
Supplement: Supplementary file 1 — ac2c03066_si_001.pdf [file ac2c03066_si_001.pdf]

Supporting Information

Protein unfolding in freeze frames: intermediate states are revealed by variable temperature ion mobility-mass spectrometry.

Jakub Ujma,[a‡] Jacquelyn Jhingree[a], Emma Norgate[a], Rosie Upton[a], Xudong Wang[a], Florian Benoit[a], Bruno Bellina[a] and Perdita Barran[a*]

[a]*Michael Barber Centre for Collaborative Mass Spectrometry*

Manchester Institute of Biotechnology, University of Manchester

131 Princess Street, M1 7DN, Manchester, United Kingdom

[‡]*Current address: Waters Corporation, Stamford Avenue, Altrincham Road, Wilmslow, SK9 4AX, UK*

*Email: perdita.barran@manchester.ac.uk

1. Ion Mobility Mass Spectrometry Methodology

1.1. Theory

A diffusion collision cross section $\Omega_d\,(T_{eff})$ is a fundamental quantity of a *two temperature kinetic theory*.[1] Throughout this report, we refer to this quantity as a collision cross section (CCS) which is a so-called *fundamental ion mobility relation.*[1–5] The expression relating ion mobility (K) to diffusion collision cross section is presented below:

$$K \approx \frac{3}{16}\frac{q}{N}\left(\frac{2\pi}{\mu k_B T_{eff}}\right)^{1/2}\frac{1}{\Omega_D(T_{eff})} \qquad (1)$$

Where q – ion charge, N – gas number density, μ – reduced mass of ion and gas molecules, k_B – Boltzmann constant. The effective ion temperature (T_{eff}) is defined as:

$$T_{eff} = T + \frac{M(KE)^2}{3k_B} \qquad (2)$$

Where T – gas temperature and E – applied electric field.

In the linear field drift tube ion mobility measurements, we determine the drift times (t_d) – the time ions require to traverse through the drift cell. Particularly in the case of using MS detection, measured time ($t_{measured}$) inevitably contains a so-called "dead time" – which corresponds to the time ions spend between the end of the drift region and the detector ($t_{measured} = t_d + t_{dead}$). Having the times measured across a range of pressure to voltage ratios (*P/V*), we can determine the mobility from the resulting linear relationship where the slope is equal to *1/K* and the dead time corresponds to the intercept.

The resolving power (*R*) is defined as a ratio of a drift time (t_d) to peaks' full width at half maximum (Δt_d). For a drift tube ion mobility separator, it depends primarily on diffusion and can be estimated from equation 3:

$$R = \frac{t_d}{\Delta t_d} = \sqrt{\frac{LEq}{2\ln 2 k_B T}} \quad (3)$$

Where *L* denotes the path length.

1.2. Experimental

Experiments were performed on a home-built ion mobility drift tube coupled to a commercial MS platform (QToF2, Micromass, Manchester, UK) as described elsewhere.[6] 50 µM solutions were electrosprayed from in-house manufactured nano-ESI tips (P-97, Sutter Instruments, Novato, USA) with the application of 1-1.5 kV. Ions were directed into a Z-spray ion source (Micromass, Manchester, UK) and subsequently into the VT IMS device. Ions were stored for 16 ms prior to release into the drift region. Pressure at the drift region was held at ~2.1 Torr (Helium). Pressure of the drift gas was monitored with a capacitance manometer (MKS Baratron). Temperature was varied by a combination of liquid nitrogen flow and resistive heating. The temperature was monitored with two platinum resistance (Pt100) thermometers immersed in the drift gas bath. In order to accurately measure the mobility of ions in helium buffer gas, arrival times were measured at several electric fields (3-14 Td) and the linear correlation ($R^2 > 0.999$) of arrival time vs *P/V* was obtained, where the slope is inversely proportional to the mobility *K* and the intercept provides the time spent in the QToF prior to detection (dead time).[7,8] A typical error in CCS due to uncertainty in measurements of pressure, temperature and the *P/V* → *1/K* fitting is < 3%.

1.3 Framework

The Framework model allows the prediction of the upper and lower CCS boundaries for proteins from their amino acid sequence. Full details of the calculations are described elsewhere. [9,10] In brief, the lower boundary is calculated by assuming the protein in its most folded, globular state occupies a spherical shape. The equation for a protein sphere is as follows where *r* is the radius of a sphere:

$$CCS_{lower}(\text{Å}^2) = \pi r^2 = \pi(\frac{3V}{4\pi})^{2/3}$$

Equation 1

The answer from Equation 1 is multiplied by a helium scaling factor of 1.19 to determine the smallest possible CCS value.

The upper boundary assumes the protein amino acid sequence is 'stretched out' so that the alpha carbons in the protein chain are as far apart as theoretically possible. This stretched out protein can be approximated as a cylinder. The equation for the upper boundary is as follows where *r* is the radius of a cylinder and *l* is the length of a cylinder:

$$CCS_{upper}(\text{Å}^2) = \left(\frac{4}{\pi}\right)rl + 2r^2$$

Equation 2

The answer from Equation 2 is multiplied by a helium scaling factor of 1.19 to determine the largest possible CCS value for a particular protein.

In order to predict low temperature framework values, we have multiplied the answers from Equations 1 and 2 by a factor of 1.07. We know from experimental work shown here and in other VT-IMS work,[6,11] on decreasing the temperature to deep-freeze temperatures (~150-180 K) the CCS increases by approximately 7 %, due to an increased effect of long range ion-molecule interactions at lower temperatures. PSA calculations at lower temperatures also predict an increase in CCS of 5% for lysozyme (295 K compared with 160 K) and 6% for ubiquitin (295 K compared with 150 K), in good agreement with experimental observations. These temperature-scaled framework values are shown as green dashed lines in Figure 3.

We are grateful to Dale Stuchfield for his insights into these calculations.

2. Additional Figures

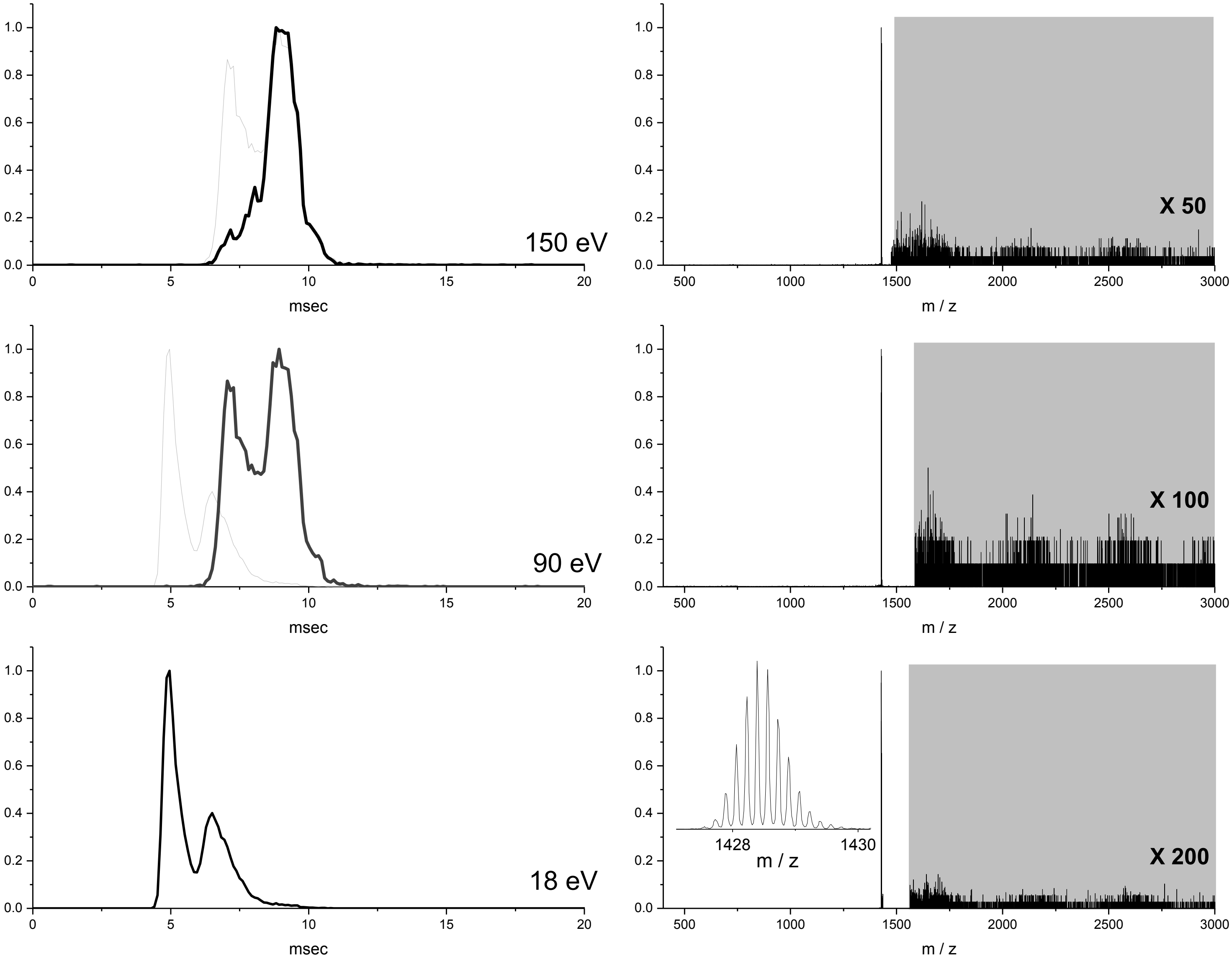

Figure S1A Activated ion mobility experiments were performed on a Waters Synapt G2Si mass spectrometer. A 10 µM ubiquitin sample was sprayed from 50 mM AmAc using a nano-ESI ion source. The instrument parameters were adjusted to prevent protein activation/unfolding and to mimic the drift time profile obtained with the VT IM-MS instrument. From the full MS spectrum, the +6 charge state of ubiquitin was m/z selected and then collisionally activated using different acceleration voltages in the trap cell region; pre mobility separation. The bottom panel (18 eV) shows the non-activated experiment data where the corresponding ATDs (LHS) and mass spectra (RHS) are shown. The middle and top panels show the data obtained with an activation of 90 eV and 150 eV, respectively. The experiments were stopped before the appearance of fragmentation ions. The grey trace represents the ATD obtained at the previous activation voltage. Whilst it is clear that the protein undergoes conformational transitions upon collisional activation, there is no evidence for any charge stripped products, fragmentation, or of dissociation from underlying multimeric species (see zoomed region of MS, RHS).

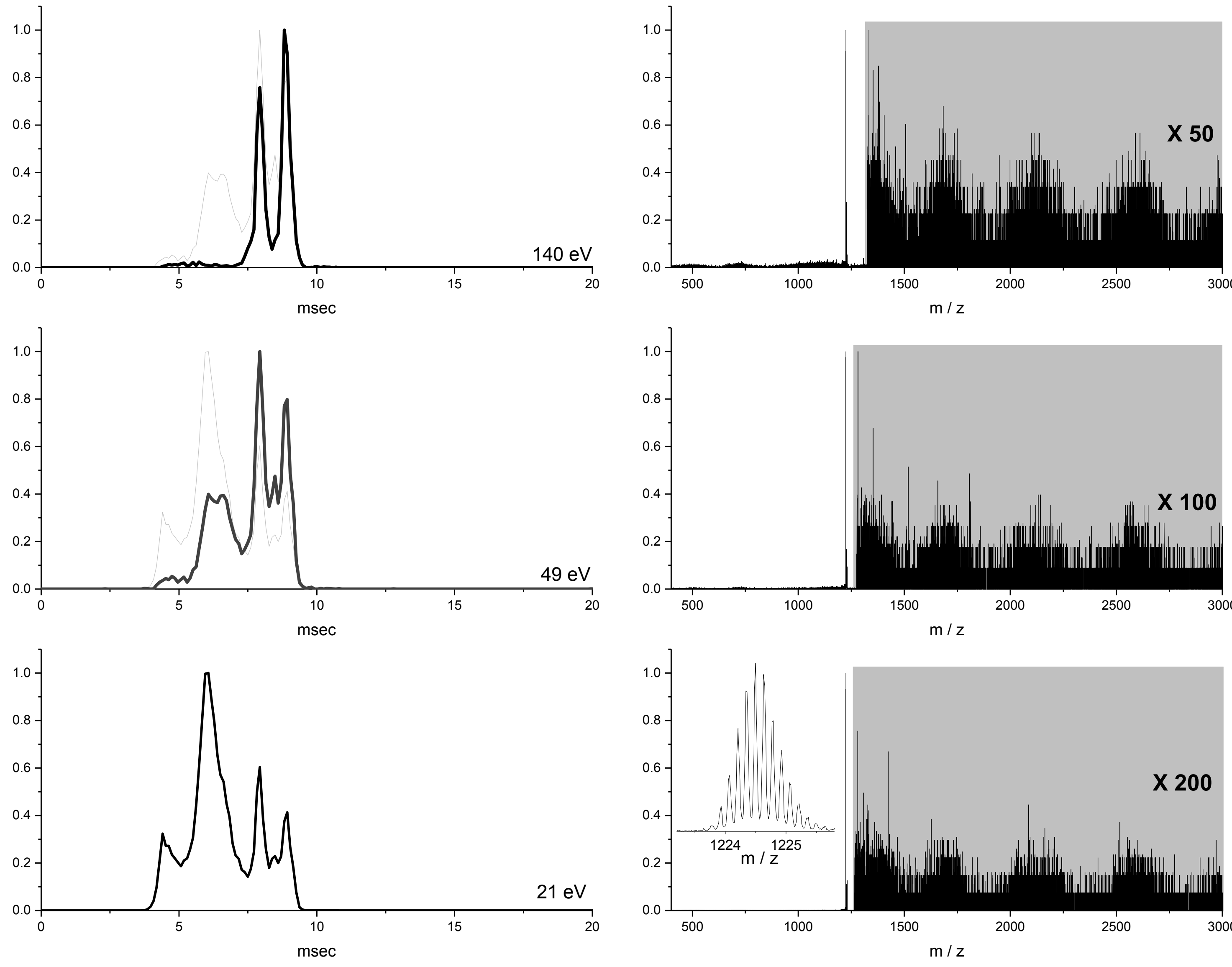

Figure S1B Activated ion mobility experiments were performed on a Waters Synapt G2Si mass spectrometer. A 10 µM ubiquitin sample was sprayed from 50 mM AmAc using a nano-ESI ion source. The instrument parameters were adjusted to prevent protein activation/unfolding and to mimic the drift time profile obtained with the VT IM-MS instrument. From the full MS spectrum, the 7+ charge state of ubiquitin was m/z selected and then collisionally activated using different acceleration voltages in the trap cell region; pre mobility separation. The bottom panel (21 eV) shows the non-activated experiment data where the corresponding ATDs (LHS) and mass spectra (RHS) are shown. The middle and top panels show the data obtained with an activation of 49 eV and 140 eV, respectively. The experiments were stopped before the appearance of fragmentation ions. The grey trace represents the ATD obtained at the previous activation voltage. Whilst it is clear that the protein undergoes conformational transitions, there is no evidence for any charge stripped products, fragmentation, or of substantial dissociation from underlying multimeric species (see zoomed region of MS, RHS).

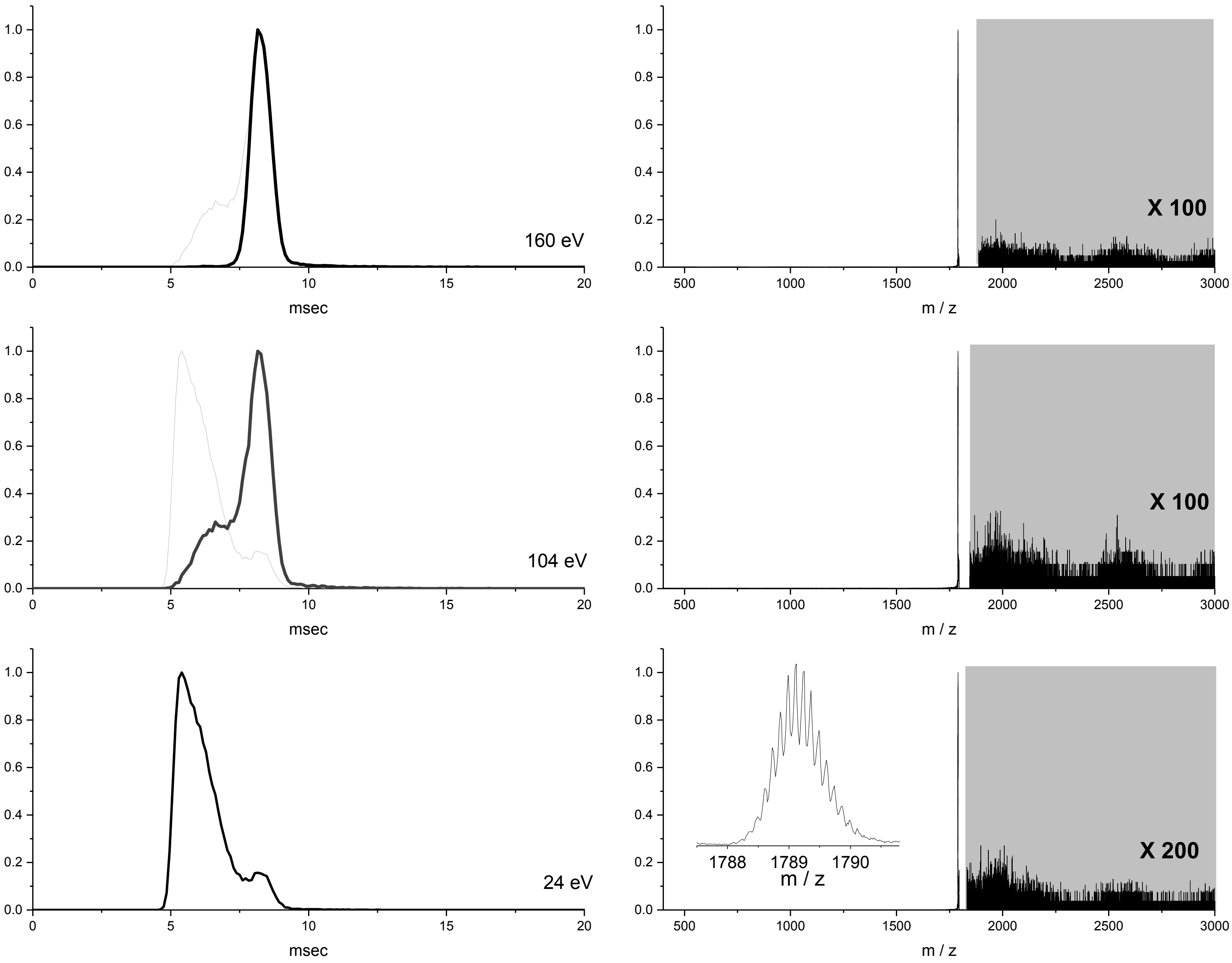

Figure S2A Activated ion mobility experiments were performed on a Waters Synapt G2Si mass spectrometer. A 10 µM lysozyme sample was sprayed from 50 mM AmAc using a nano-ESI ion source. The instrument parameters were adjusted to prevent protein activation/unfolding and to mimic the drift time profile obtained with the VT IM-MS instrument. From the full MS spectrum, the +8 charge state of lysozyme was m/z selected and then collisionally activated using different acceleration voltages in the trap cell region; pre mobility separation. The bottom panel (24 eV) shows the non-activated experiment data where the corresponding ATDs (LHS) and mass spectra (RHS) are shown. The middle and top panels show the data obtained with an activation of 104 eV and 160 eV, respectively. The experiments were stopped before the appearance of fragmentation ions. The grey trace represents the ATD obtained at the previous activation voltage. Whilst it is clear that the protein undergoes a conformational transition, there is no evidence for any charge stripped products, fragmentation, or of substantial dissociation from underlying multimeric species (see zoomed region of MS, RHS).

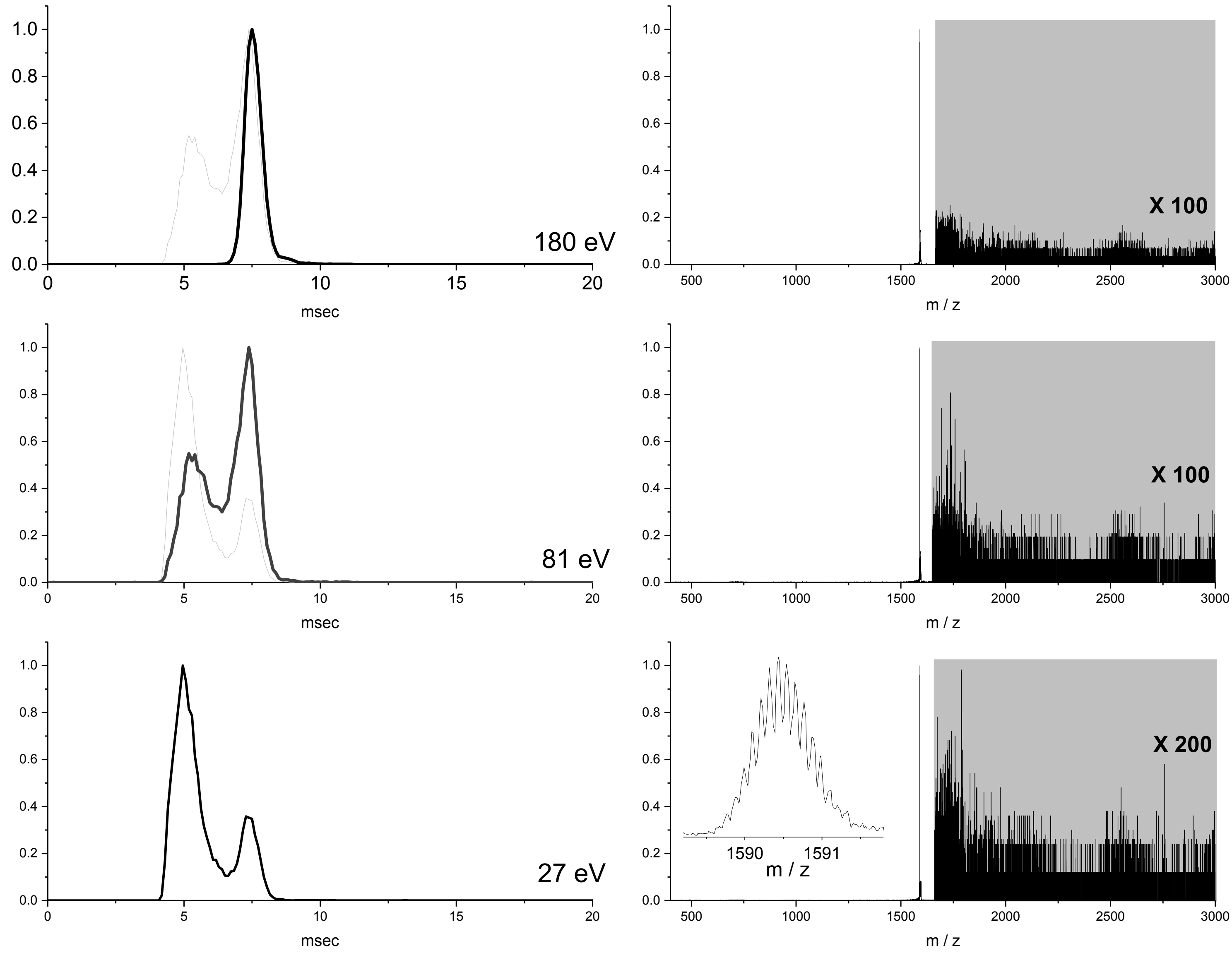

Figure S2B Activated ion mobility experiments were performed on a Waters Synapt G2Si mass spectrometer. A 10 µM lysozyme sample was sprayed from 50 mM AmAc using a nano-ESI ion source. The instrument parameters were adjusted to prevent protein activation/unfolding and to mimic the drift time profile obtained with the VT IM-MS instrument. From the full MS spectrum, the +9 charge state of lysozyme was m/z selected and then collisionally activated using different acceleration voltages in the trap cell region; pre mobility separation. The bottom panel (27 eV) shows the non-activated experiment data where the corresponding ATDs (LHS) and mass spectra (RHS) are shown. The middle and top panels show the data obtained with an activation of 81 eV and 180 eV, respectively. The experiments were stopped before the appearance of fragmentation ions. The grey trace represents the arrival time distribution obtained at the previous activation voltage. Whilst it is clear that the protein undergoes a conformational transition, there is no evidence for any charge stripped products, fragmentation, or of substantial dissociation from underlying multimeric species (see zoomed region of MS, RHS).

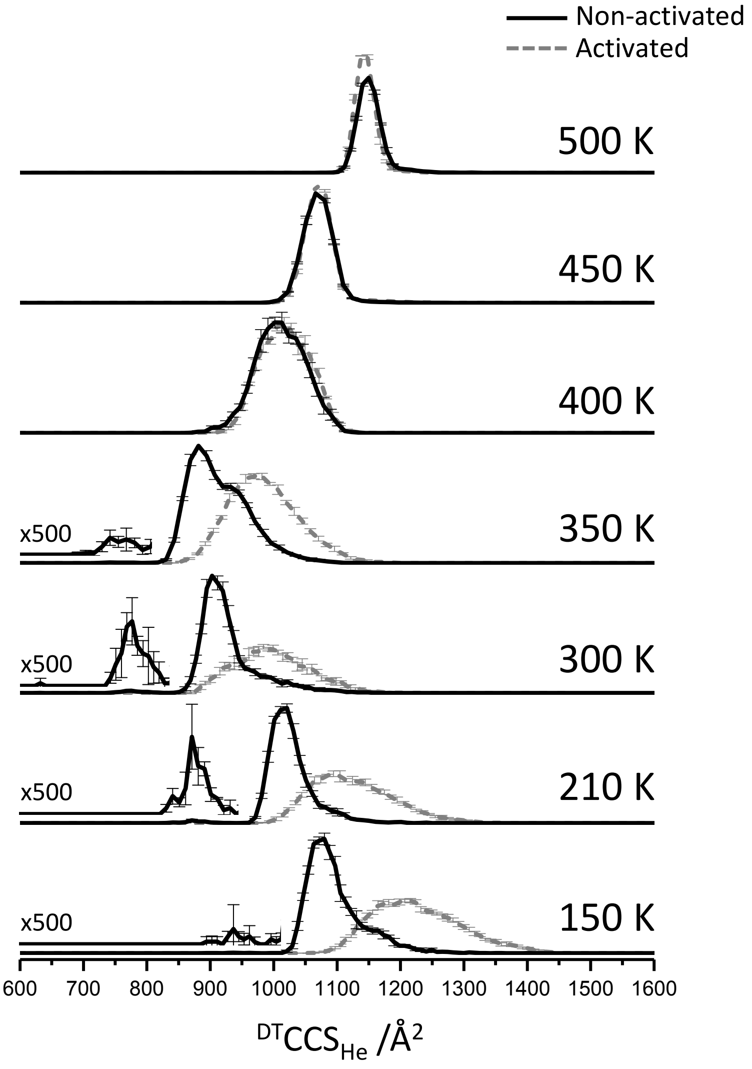

Figure S3. Collision Cross Section distributions of ubiquitin 5+ sprayed from 50 µM solution in 50 mM ammonium acetate pH 6.8. Solid lines represent data obtained with no in-source activation at a range of temperatures (150-500 K). Dashed lines represent the respective data recorded with the in-source activation on. Error bars correspond to standard deviation from three, 1 minute long acquisitions. At temperatures 150-350 K, a small amount of dimer was detected which is highlighted with the additional traces, where intensity is scaled by a factor of 500. Dimeric species were not observed for +6 ions. Activated data has been normalized to the area of the non-activated data.

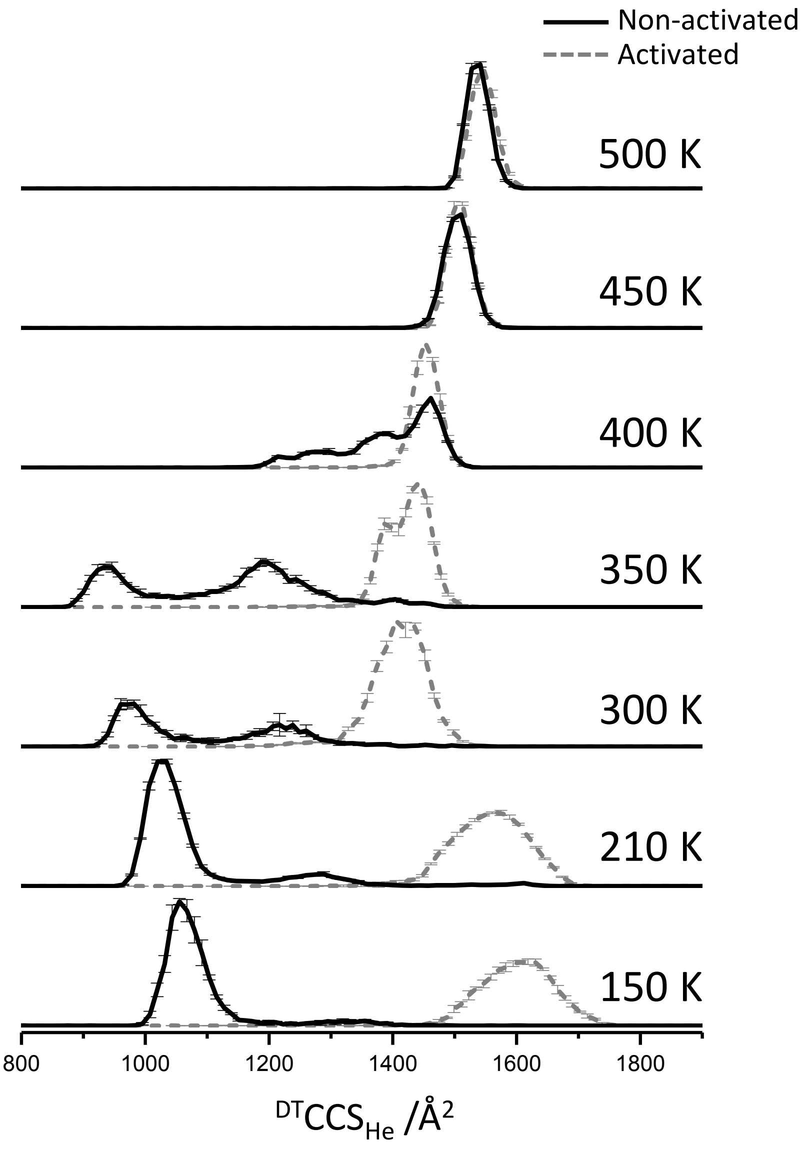

Figure S4. Collision Cross Section distributions of ubiquitin 7+ sprayed from 50 µM solution in 50 mM ammonium acetate pH 6.8. Solid lines represent data obtained with no in-source activation at a range of temperatures (150- 500 K). Dashed lines represent the respective data recorded with the in-source activation on. Error bars correspond to standard deviation from three, 1 minute long acquisitions. Activated data has been normalized to the area of the non-activated data.

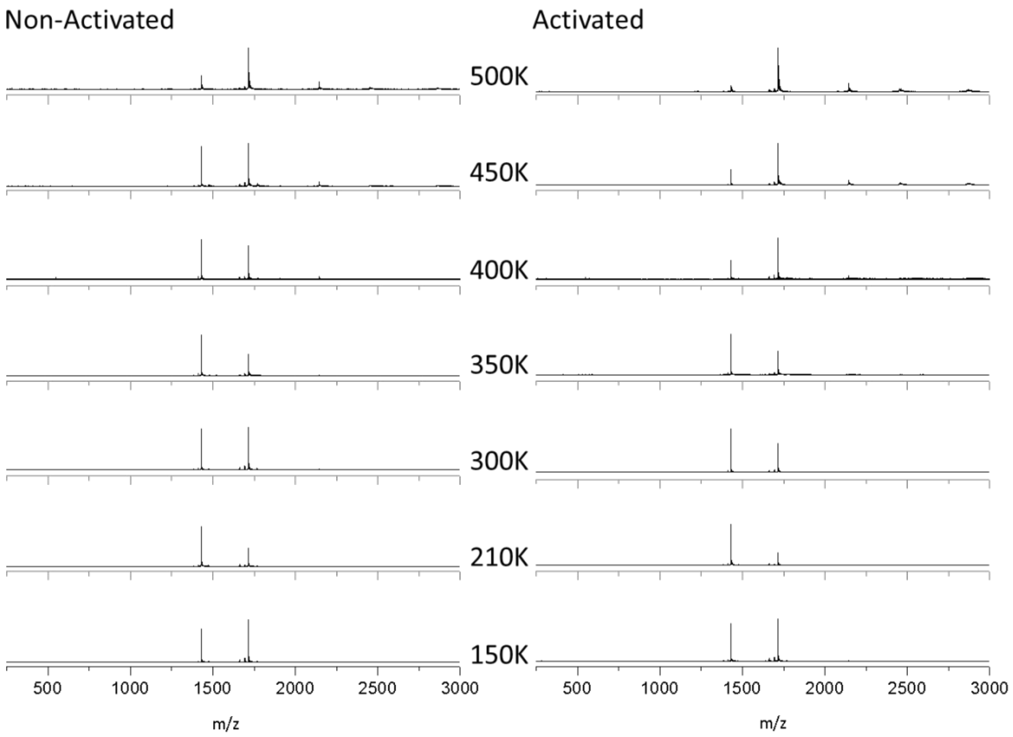

Figure S5. Mass spectra of ubiquitin sprayed at a concentration of 50 µM solution in 50 mM ammonium acetate, pH 6.8. Left: data obtained with no in-source activation at a range of temperatures (150-500 K). Right: the respective data with in-source activation on. Differences in intensities between charge states 5+ and 6+ over 150-400 K reflect the variations in day-to-day fine tuning of the n-ESI source and ion buncher settings. Charge reduction is observed at temperatures 450-500 K, we are yet uncertain of the exact origin of this phenomenon.

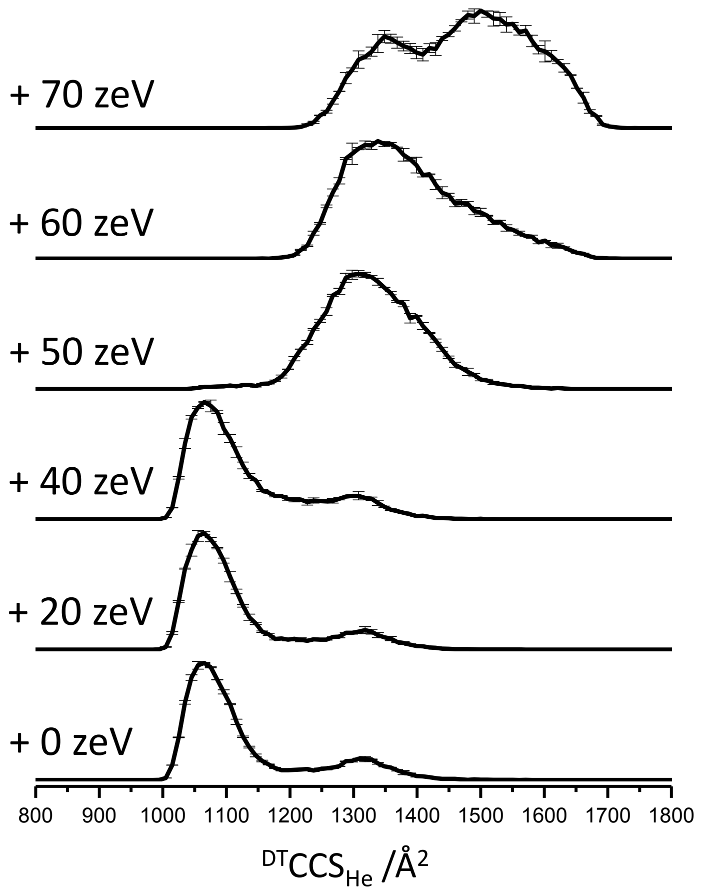

Figure S6. Collision Cross Section distributions of ubiquitin 6+ sprayed from 50 µM solution in 50 mM ammonium acetate pH 6.8 at a range of in-source collision energies recorded at 150 K. Error bars correspond to standard deviation from two, 1 minute long acquisitions.

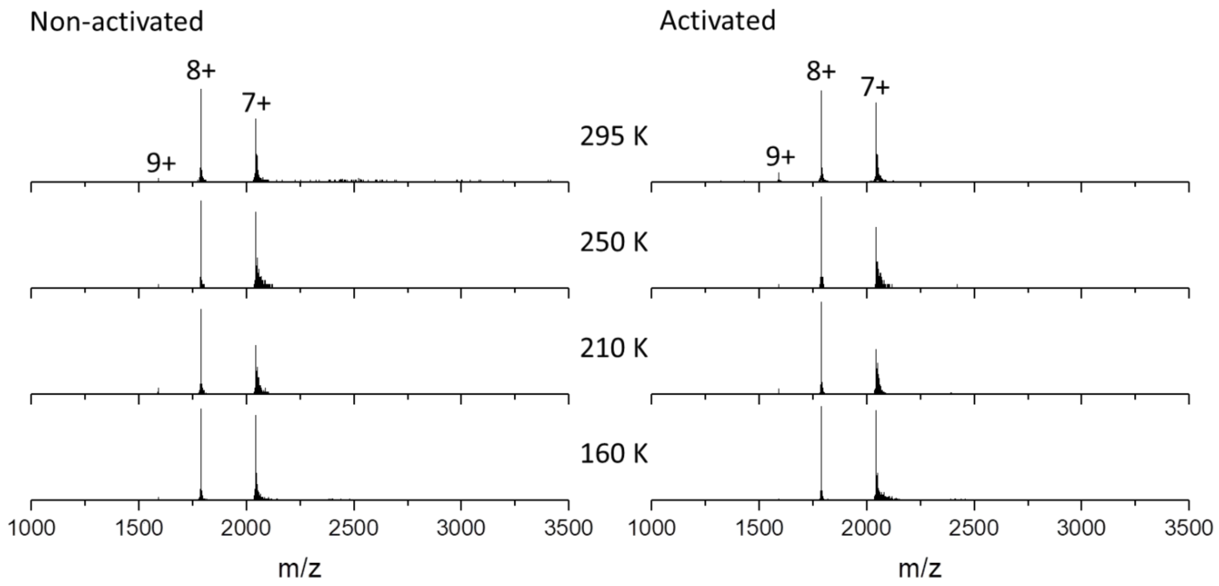

Figure S7. Mass spectra of lysozyme sprayed at a concentration of 30 µM solution in 50 mM ammonium acetate, pH 6.8. Left: data obtained with no in-source activation at a range of temperatures (160- 295 K). Right: the respective data with in-source activation on. Differences in intensity ratio between charge states 7+ and 8+ reflect the variations in day-to-day fine tuning of the n-ESI source and ion buncher settings.

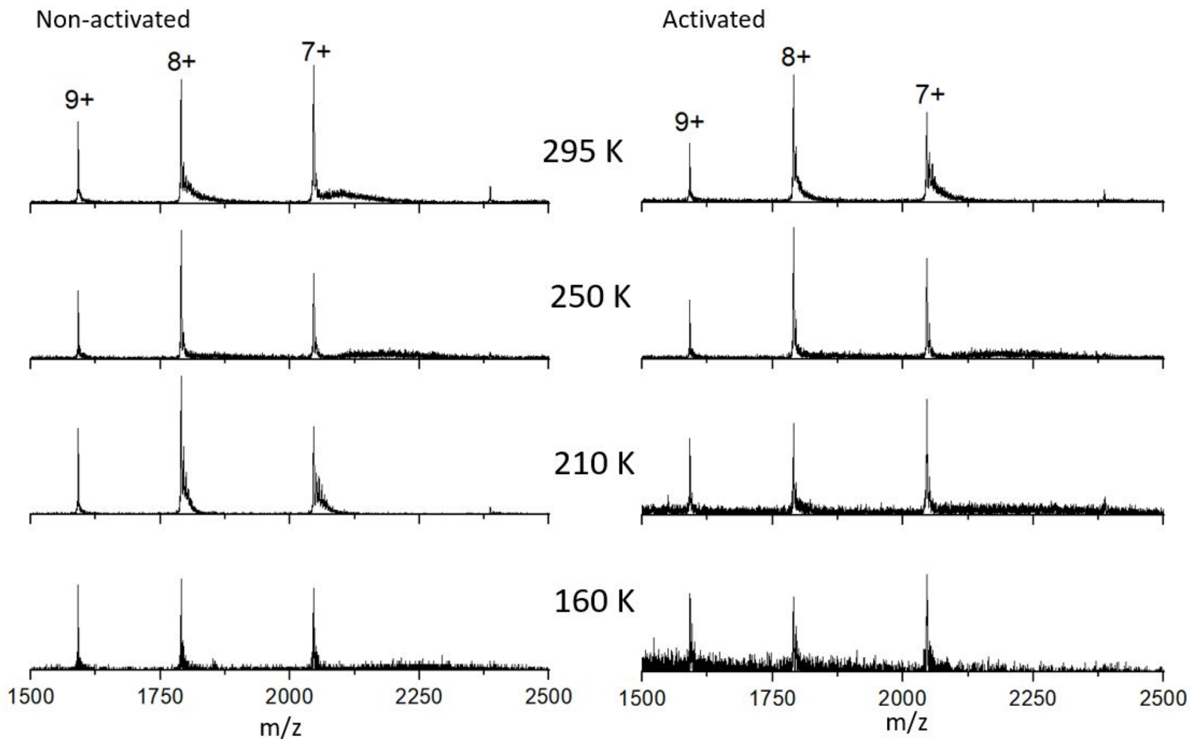

Figure S8 Mass spectra of disulphide-reduced lysozyme sprayed at a concentration of 30 µM solution 50 mM ammonium acetate and 10mM DTT, pH 6.8. Left: data obtained with no in-source activation at a range of temperatures (160- 295 K). Right: the respective data with in-source activation on. Differences in intensity ratio between charge states 7+ and 8+ reflect the variations in day-to-day fine tuning of the n-ESI source.

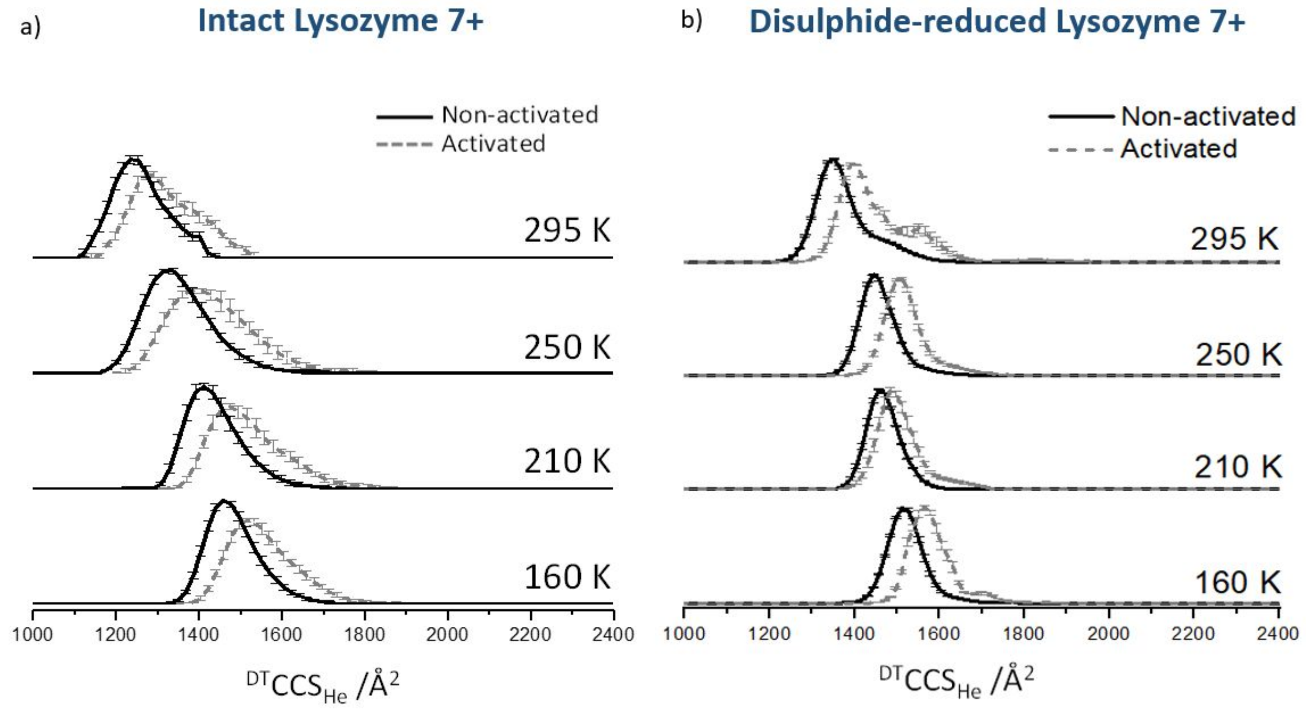

Figure S9. Collision Cross Section distributions of lysozyme (a) and disulphide-reduced lysozyme (b) 7+ sprayed from 30 µM solution in 50 mM ammonium acetate pH 6.8. Disulphide-reduced lysozyme solution additionally contains 10mM DTT. Solid lines represent data obtained with no in-source activation at a range of temperatures (160- 295 K). Dashed lines represent the respective data recorded with the in-source activation on. Error bars correspond to standard deviation from six, 30 second long acquisitions. Activated data has been normalized to the area of the non-activated data.

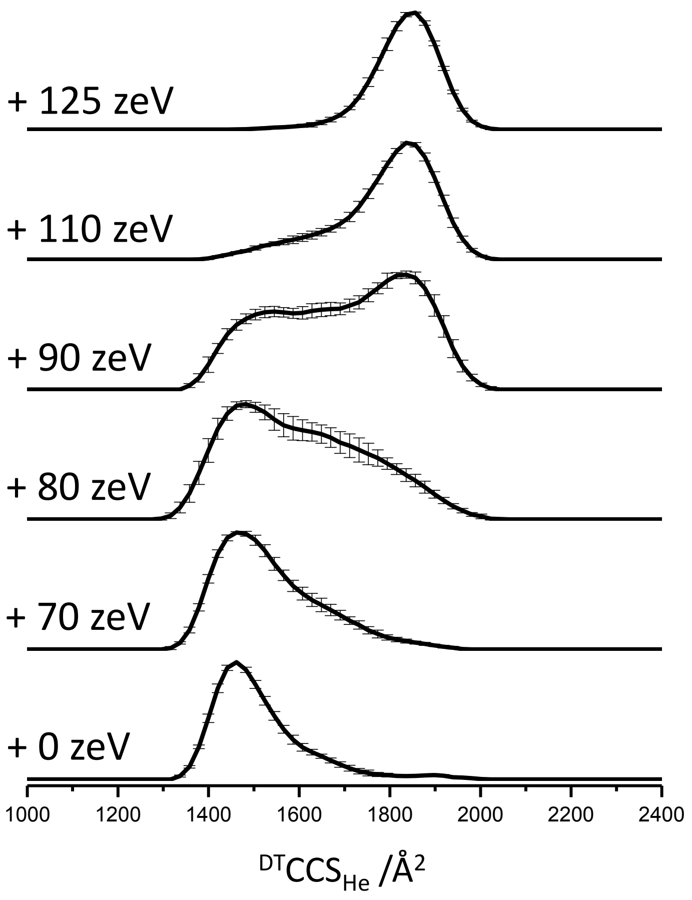

Figure S10. Collision Cross Section distribution of lysozyme 8+ sprayed from 30 µM solution in 50 mM ammonium acetate pH 6.8 at a range of in-source collision energies recorded at 160 K. Error bars correspond to standard deviation from six, 30 second long acquisitions.

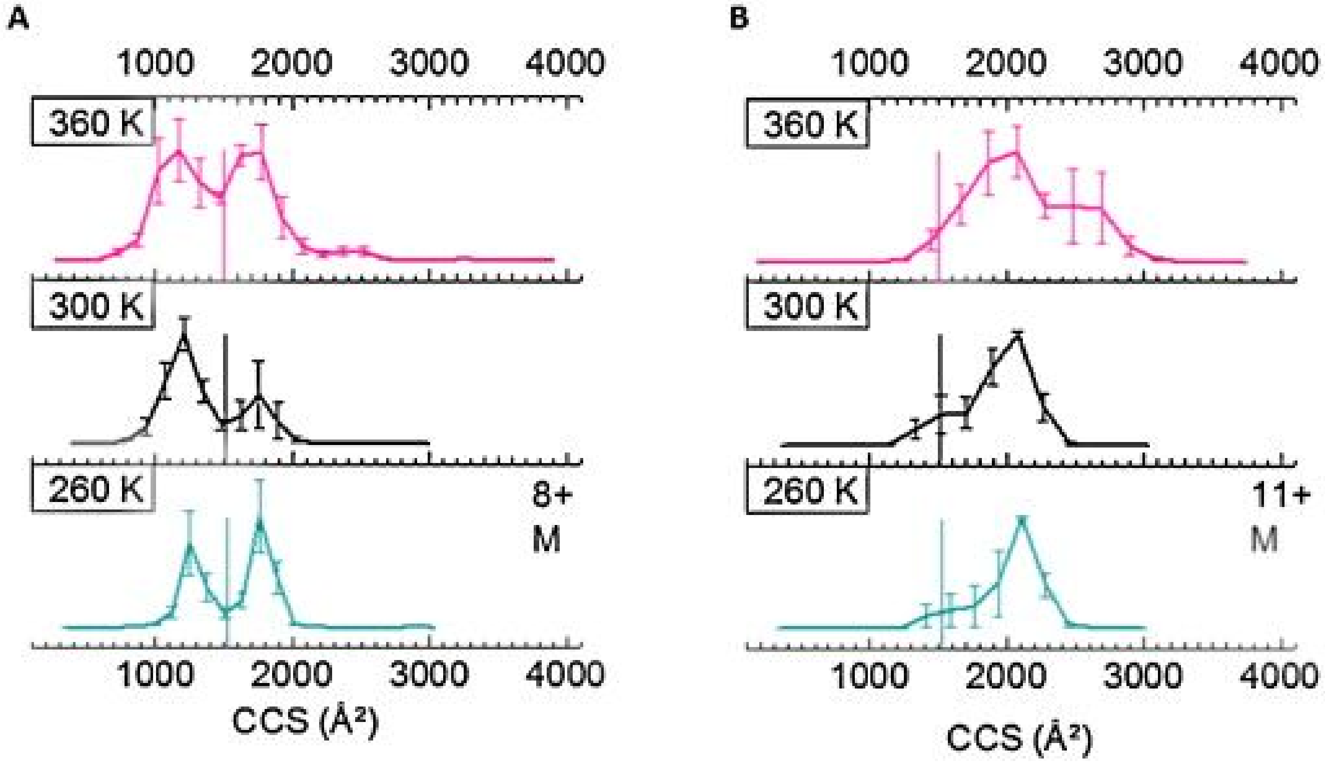

Figure S11. VT IM-MS Collision Cross Section distributions of intact lysozyme 8+ and 11+ as reported by us previously[12] sprayed from 50 µM solution with 40-fold molar excess of sodium iodide (2 mM), on instrument 2 detailed in main paper, at 360, 300 and 260K to compare with data shown in Figure 2. Figure reproduced with permission.

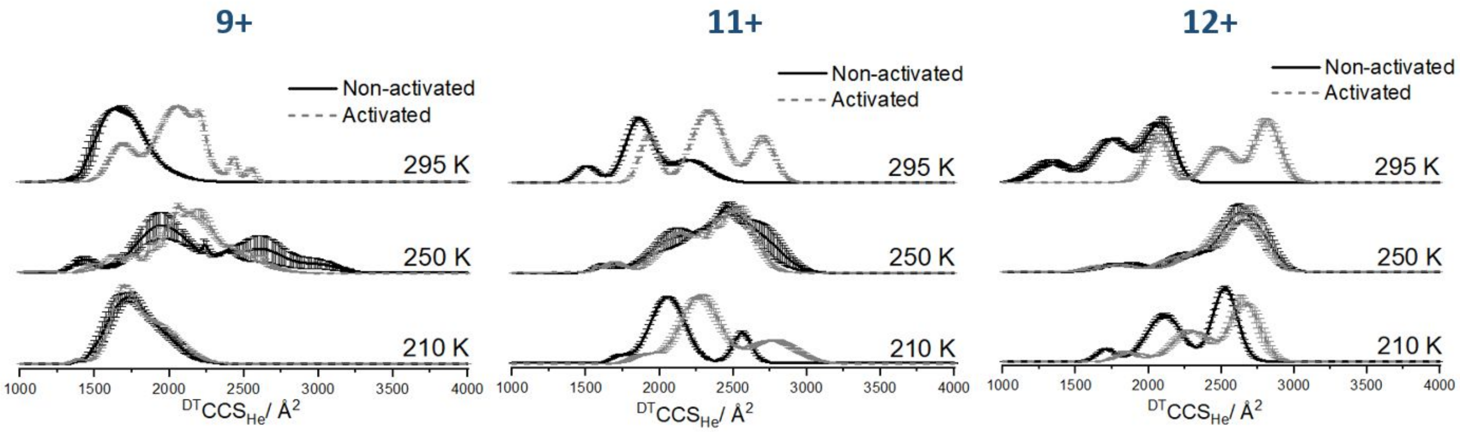

Figure S12 Collision Cross Section distributions of alpha-synuclein charge states 9+, 11+ and 12+ sprayed from 20 µM solution in 100 mM ammonium acetate. Solid lines represent data obtained with no in-source activation at a range of temperatures (210- 295 K). Dashed lines represent the respective data recorded with the in-source activation on. Error bars correspond to standard deviation from five, 30 second long acquisitions.

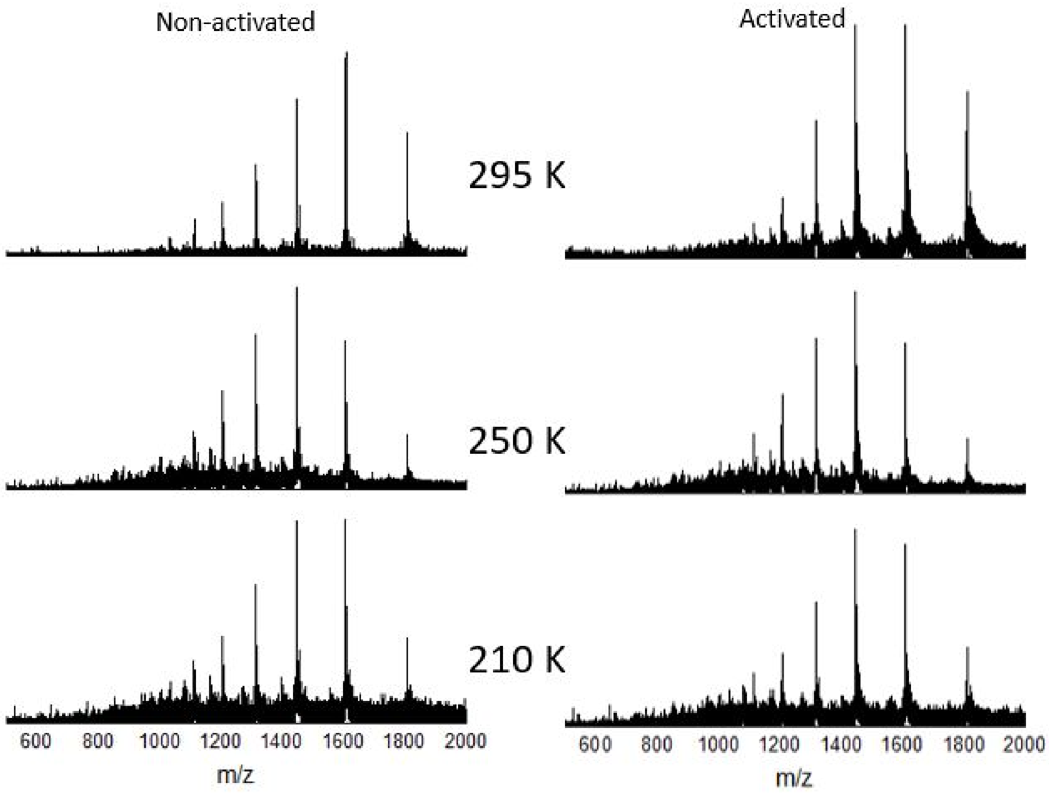

Figure S13 Mass spectra of alpha-synuclein sprayed at a concentration of 10 µM solution in 100 mM ammonium acetate. Left: data obtained with no in-source activation at a range of temperatures (210-295 K). Right: the respective data with in-source activation on. Differences in intensity ratio between charge states reflect the variations in day-to-day fine tuning of the n-ESI source and ion buncher settings.

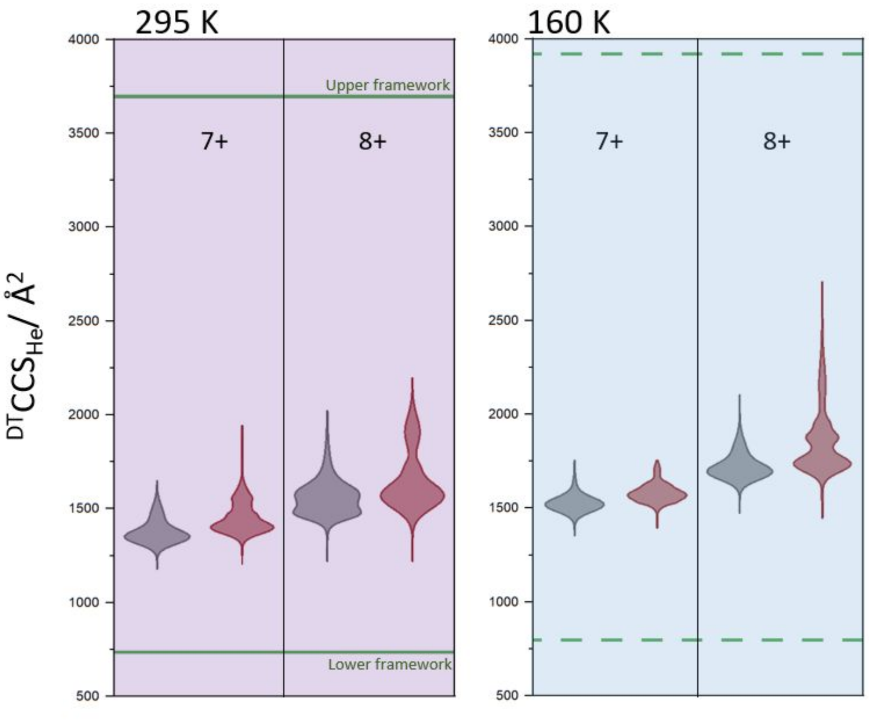

Figure S14: Summary of experimental CCS distributions for disulfide-reduced lysozyme 7 and 8+ for both non-activated (grey violin plots) and activated (red violin plots).

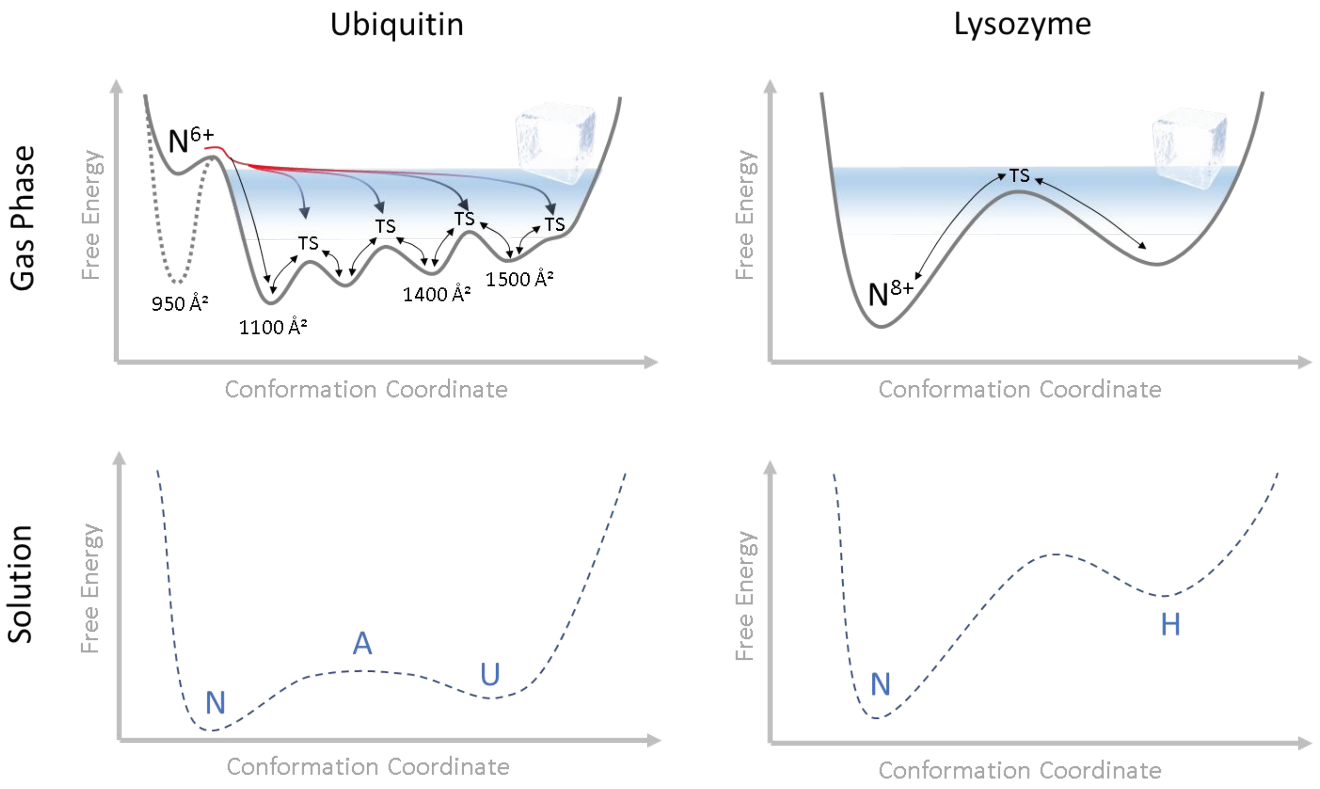

Figure S15 Hypothetical 1D gas-phase folding free energy surfaces of ubiquitin and lysozyme in the gas phase and in solution.

3. References

(1) Viehland, L. A.; Mason, E. A. Gaseous Ion Mobility and Diffusion in Electric Fields of Arbitrary Strength. *Ann. Phys. (N. Y).* **1978**, *110* (2), 287–328. https://doi.org/10.1016/0003-4916(78)90034-9.

(2) Langevin, M. P. Une Formule Fondamentale De Theorie Cinetique. *Ann. Chim. Phys.* **1905**, *8*, 245–277.

(3) Revercomb, H. E.; Mason, E. A. Theory of Plasma Chromatography/Gaseous Electrophoresis. Review. *Anal. Chem.* **1975**, *47* (7), 970–983. https://doi.org/10.1021/ac60357a043.

(4) Mason, E. A.; McDaniel, E. *Transport Properties of Ions in Gases*; 1988.

(5) Siems, W. F.; Viehland, L. A.; Hill, H. H. Improved Momentum-Transfer Theory for Ion Mobility. 1. Derivation of the Fundamental Equation. *Anal. Chem.* **2012**, *84*, 9782–9791.

(6) Ujma, J.; Giles, K.; Morris, M.; Barran, P. E. New High Resolution Ion Mobility Mass Spectrometer Capable of Measurements of Collision Cross Sections from 150 to 520 K. *Anal. Chem.* **2016**, *88*, 9469–9478. https://doi.org/10.1021/acs.analchem.6b01812.

(7) Kemper, P. R.; Bowers, M. T. A Hybrid Double-Focusing Mass Spectrometer-High-Pressure Drift Reaction Cell to Study Thermal Energy Reactions of Mass-Selected Ions. *J. Am. Soc. Mass Spectrom.* **1990**, *1*, 197–207. https://doi.org/10.1016/1044-0305(90)85036-L.

(8) Wyttenbach, T.; Bowers, M. Design of a New Electrospray Ion Mobility Mass Spectrometer. *Int J Mass Spectrom* **2001**, *212* (1–3), 13–23.

(9) Beveridge, R.; Covill, S.; Pacholarz, K. J.; Kalapothakis, J. M. D.; Macphee, C. E.; Barran, P. E. A Mass-Spectrometry-Based Framework To Define the Extent of Disorder in Proteins. *Anal. Chem.* **2014**, *86* (22), 10979–10991.

(10) Beveridge, R.; Migas, L. G.; Kriwacki, R. W.; Barran, P. E. Ion Mobility Mass Spectrometry Measures the Conformational Landscape of P27 and Its Domains and How This Is Modulated upon Interaction with Cdk2/Cyclin A. *ChemRxiv* **2018**, No. November. https://doi.org/10.26434/chemrxiv.7303988.

(11) Norgate, E. L.; Upton, R.; Hansen, K.; Bellina, B.; Brookes, C.; Politis, A.; Barran, P. E. Cold Denaturation of Proteins in the Absence of Solvent. *Preprint* **2021**.

(12) Berezovskaya, Y.; Porrini, M.; Barran, P. E. The Effect of Salt on the Conformations of Three Model Proteins Is Revealed by Variable Temperature Ion Mobility Mass Spectrometry. *Int. J. Mass Spectrom.* **2013**, *345–347*, 8–18. https://doi.org/10.1016/j.ijms.2013.02.005.
